# Antibiotic Prescribing in DR Congo: A Knowledge, Attitude and Practice Survey among Medical Doctors and Students

**DOI:** 10.1371/journal.pone.0055495

**Published:** 2013-02-18

**Authors:** Kamala Thriemer, Yves Katuala, Bibi Batoko, Jean-Pierre Alworonga, Hugo Devlieger, Christel Van Geet, Dauly Ngbonda, Jan Jacobs

**Affiliations:** 1 Department of Clinical Sciences, Institute of Tropical Medicine, Antwerp, Belgium; 2 Department of Pediatrics, University Hospital Kisangani, Kisangani, Democratic Republic of the Congo; 3 Department of Pediatrics, University Hospital Leuven, Leuven, Belgium; University of Texas HSC at San Antonio, United States of America

## Abstract

**Objectives:**

Antibiotic resistance (ABR) particularly hits resource poor countries, and is fuelled by irrational antibiotic (AB) prescribing. We surveyed knowledge, attitudes and practices of AB prescribing among medical students and doctors in Kisangani, DR Congo.

**Methods:**

Self-administered questionnaires.

**Results:**

A total of 184 questionnaires were completed (response rate 94.4%). Knowledge about AB was low (mean score 4.9/8 points), as was the estimation of local resistance rates of *S.* Typhi and *Klebsiella* spp.(correct by 42.5% and 6.9% of respondents respectively). ABR was recognized as a problem though less in their own practice (67.4%) than nation- or worldwide (92.9% and 85.5%, p<.0001). Confidence in AB prescribing was high (88.6%) and students consulted more frequently colleagues than medical doctors when prescribing (25.4% versus 11.6%, p  = 0.19). Sources of AB prescribing included pharmaceutical companies (73.9%), antibiotic guidelines (66.3%), university courses (63.6%), internet-sites (45.7%) and WHO guidelines (26.6%). Only 30.4% and 16.3% respondents perceived AB procured through the central procurement and local pharmacies as of good quality. Local AB guidelines and courses about AB prescribing are welcomed (73.4% and 98.8% respectively).

**Conclusions:**

This data shows the need for interventions that support rational AB prescribing.

## Introduction

Increasing resistance of bacterial pathogens to commonly used antibiotics (AB) has become a world-wide public health concern [1]. Spread of antibiotic resistance (ABR) is causing not only increased morbidity and mortality but also a high economic burden. Health systems in low income countries which are already struggling with chronic underfunding and weak institutional structures are particularly hit by ABR [1].

The causes that drive ABR in low income countries are well known. The main factors are irrational drug use such as over-prescription and unnecessary prescription of AB (such as for viral infections), incomplete treatments and self-medication as well as insufficient infection control measures to prevent spread of resistant bacteria both in the community and the hospital [2]. The prescribing behavior of medical doctors plays a key role in the consumption of AB and is a potential tool for control and containment of ABR. Factors thriving prescribing behavior of medical doctors can be analyzed by so-called KAP-surveys, in which knowledge (K), attitudes (A) and practices (P) of prescribers are assessed. In addition, medical students are an important target group for sustainable AB prescribing intervention programs [3].

There is only little KAP-information among prescribers in hospital settings [4], [5], [6], [7], [8], [9] compared to a wider variety of publications reporting from community settings. In addition, the available studies have all been conducted in high (Europe, U.S.) or middle (Brazil, Peru) income countries. We therefore conducted a KAP-survey about AB prescribing among last year medical students and medical doctors in a low income setting in Central Africa.

## Methods

### Study Design and Setting

**Figure 1:**
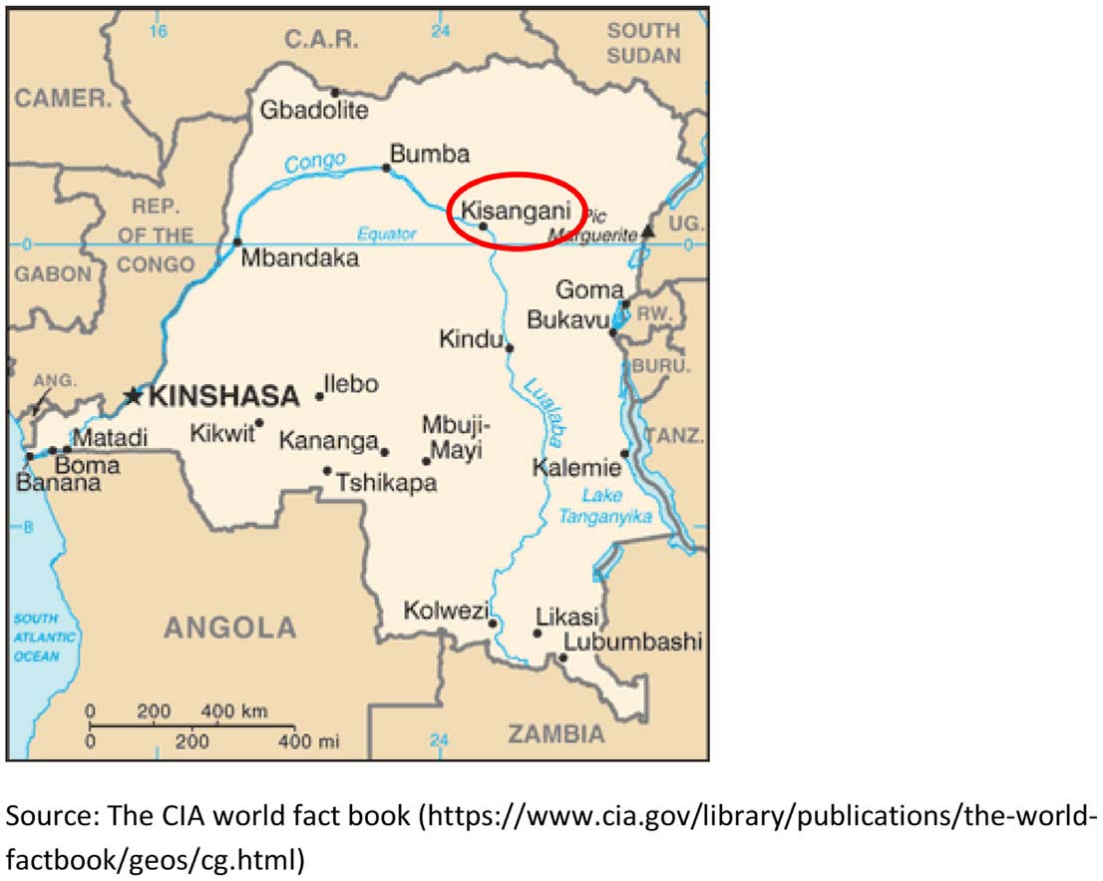
Map of the study site.

The survey was conducted during March – April 2011 in the area of Kisangani, a town of 600,000 inhabitants in the Oriental Province, in the center of Democratic Republic of the Congo (DRC) (Figure 1). Kisangani comprises a health district with five health zones. In addition to the public health centers (one for each health zone), there are numerous private and confessional health centers in the area.

The University Hospital of Kisangani (UH Kisangani) is a 129-bed referral and teaching hospital accommodating students of the Faculty of Medicine of the University of Kisangani for clinical clerkships. Despite on top of the health pyramid, UH Kisangani is only basically equipped as a consequence of recent wars and a degraded economic situation. There are no functioning microbiology services and no antibiotic committee. Bed occupancy rate in 2011 was estimated at 45.5% (UK Kisangani, annual report 2011). The other health centers and the two referral hospitals in Kisangani have similar constraints of infrastructure and equipment.

### Respondents

This study was performed as a cross-sectional survey with purposeful sampling among last year medical students (who are prescribing as part of their practice) as well as prescribing medical doctors with different extent of work experience. The survey was a self-administered questionnaire which was distributed and collected by trained collaborators (co-authors Y.K. and B.B.).

Last year medical students were recruited at UH Kisangani (total number of students was 131). Prescribing medical doctors were visited on-site at the hospitals or health centers after having made an appointment. Medical doctors working in the administrative services were not addressed. According to the Provincial Health Division, the total number of prescribing medical doctors in Kisangani in 2011 was estimated at 185 (personal communication).

The surveys were given to the respondents who filled in the forms immediately. During completion, the collaborator was available for explanation. The filled-in copies were handed over immediately after completion. No incentives were given to the respondents.

### Survey Instrument

The survey instrument was developed according to a previous questionnaire used in Peru [9], and adapted to the setting in Kisangani. Translation from the original Spanish version into French was performed by one of the authors (J.J.)and two local experts (B.B. and J.A.) carefully reviewed the translation for compliance of phrasing and wording with local French and professional language. Prior to release, the survey was validated for content and relevance by a team of four professionals (D.N., A.J., H.D and B.B).

The original questionnaire was amended with questions addressing the resources about AB prescribing (including pharmaceutical companies), the perceptions of the causes of ABR and the quality of locally available AB (one question each). The final survey consisted of 33 questions (Table S1). Three questions addressed work experience and prescription patterns (n  = 3), awareness on the current scope of ABR (n  = 5), the respondents’ confidence in AB prescribing and willingness to seek input from superiors or peers (n  = 3). In addition, information was collected about the sources of information used and continuing education about AB prescribing (n  = 2 subdivided questions ) and about factors influencing the decision about AB prescribing (n  = 7). Acceptability and appropriateness of potential interventions were assessed with another three questions (n = 3). Finally, knowledge was assessed in a total of eight questions (n = 8), divided in three case presentations and five questions about AB properties. A knowledge score was calculated as the sum of correct answers to these questions. Two additional knowledge questions addressed the respondents’ estimations of local resistance rates of two key-pathogens (n = 2), but where not included in the overall score.

### Ethical Considerations

Pending the installation of an ethical committee in the Oriental Province and at the UH Kisangani, the study was assessed and approved by the Provincial Health Officer and the Director’s Board of UH Kisangani, the highest possible authority. All data was analyzed anonymously.

### Data Registration and Statistical Analyses

Data were anonymously entered in an Excel database (Microsoft Corporation, Redmond, Washington, USA). Proportions and means were calculated for categorical and continuous variables respectively. Chi square, Fisher's exact test and Mann-Whitney U test were performed as appropriate to calculate a 95% level of significance. All statistical analyses was performed using STATA version 12 (Statacorp Texas, USA).

## Results

### Baseline Data: Coverage, Response Rate, Professional Profile

Eighty-nine of the estimated 185 medical doctors in Kisangani were addressed, as well 106 of the 131 medical students at UH Kisangani (coverage rates of 48.1% and 80.9% respectively). Of the total of 195 questionnaires, 11 (5.6%) were either not returned or incompletely filled in (all from medical doctors), leaving 184 eligible surveys (response rate 94.4%). Medical doctors and students accounted for 78 (42.3%) and 106 (57.6%) of respondents respectively.

Among the medical doctors, 11.5% (n  = 9) had less than one year work experience, 38.5% (n  = 30) had between 1 and 3 year work experience, 23.1% (n  = 18) between 4 to 6 years work experience and the remaining 26.9% (n  = 21) had more than 10 years of work record. Unless otherwise stated, the results are presented for all respondents (medical students and medical doctors regardless of work experience) combined, except when there were differences between these groups.

All respondents prescribed AB to in- as well as outpatients. More than half (100/183, 54.4% - one respondent did not reply to this question) reported to prescribe AB more than once a day, 15.2% (n  = 28) and 17.4% (n  = 32) reported 1 to 2 times and 3 to 5 times per week respectively. As expected, medical students prescribed less frequently compared to medical doctors (≥1 prescription/day: 42.5% versus 85.7% and <1 prescription/day: 57.5% versus 14.3%; p<.0001).

### Knowledge

On a score of 8, the mean (± S.D.) and median (range) scores were 4.9 (±0.09) and 5 (2–8) respectively. A total of 1 (0.5%) and 20 (10.9%) of respondents scored 8 and 7 points respectively; scores of 6 and 5 were achieved by 36 (19.6%) and 55 (29.9%) respondents respectively. The remaining respondents (n  = 72, 39.1%) had a score of 4 or less. There was no significant difference in the mean score according to years of experience (p  = .19), frequency of consulting colleagues before deciding about AB prescribing (p  = .21) or previous training about AB prescribing (p  = .54).

As to the individual questions (Table 1), the results were as follows: although the majority (89.7%) of respondents correctly answered to install no AB treatment in case of non-febrile diarrhea, less than one third (27.9%) replied so to the question about upper respiratory tract infection (URTI). Further, less than 20% of respondents correctly replied to questions about dose reduction in case of renal failure and less than 40% about cross-resistance of methicillin resistant *Staphylococcus aureus* (MRSA) to beta-lactam AB. Compared to medical doctors, medical students answered three questions significantly more frequently incorrect: they more frequently advised AB in case of URTI, were less aware of the cross-resistance of MRSA and were more unsure about safe antibiotics in pregnancy.

**Table 1:** Knowledge questions and results.

No.	Question and *possible answers (correct answer in bold)*	Overall N (%) answered correctly*	N (%) of doctors answered correctly*	N (%) of students answered correctly*	p
1	A 4-year-old girl has diarrhea for 4 days (3 stools/day). She has no fever at examination nor duringthe last few days. Which treatment do you propose?				
	* a) amoxicillin p.o.*	165/184	68/78	97/106	0.340
	* b) TMP/SMX* ** *p.o.*	(89.7%)	(85.9%)	(74.5%)	
	* c) amoxicillin-clavulanic acid p.o.*				
	*** d) no antibiotic treatment, only oral rehydration***				
2	A 6-year-old child has a fever of 38°C, purulent rhinitis and angina for two days. At inspection,the throat is reddish. Which treatment do you recommend?				
	* a) TMP/SMX* ** *p.o.*	51/183	28/78	23/105	0.038
	* b) amoxicillin p.o.*	(27.9%)	(35.9%)	(21.9%)	
	* c) amoxicillin-clavulanic acid p.o.*				
	*** d) no antibiotic***				
3	During your ward round, you see two patients with severe renal failure. Patient A is a 68-year-old man suffering from serious cellulitis at the leg, he is treated with clindamycin. Patient B is a 64-year-olddiabetic woman which is blindly (empirically) treated for septicemia with ceftriaxone. Dosage reductionis needed for:				
	* a) Patient A*	34/183	12/77	22/106	0.375
	* b) Patient B*	(18.6%)	(15.6%)	(20.7%)	
	* c) both patients*				
	*** d) in neither patient A nor patient B***				
4	Which one of the following antibiotics is safe during pregnancy?				
	*** a) amoxicillin***	170/183	76/78	94/105	0.039
	* b) ciprofloxacin*	(92.9%)	(97.4%)	(89.5%)	
	* c) gentamicin*				
5	Which one of the following antibiotics has the best activity against anaerobes?				
	* a) ciprofloxacin*	171/182	72/77	99/105	0.827
	*** b) metronidazole***	(94.0%)	(93.5%)	(94.3%)	
	* c) cotrimoxazole*				
6	Methicillin resistant *- Staphylococcus aureus* is susceptible to:				
	* a) amoxicillin-clavulanic acid*	64/174	33/73	31/101	0.0500
	* b) cefotaxime*	(36.8%)	(45.2%)	(30.7%)	
	* c) ceftriaxone*				
	*** d) none of those antibiotics***				
7	Which one of the following antibiotic most effectively crosses the blood-brain barrier?				
	* a) clindamycin*	132/179	58/75	74/104	0.354
	*** b) ceftriaxone***	(73.7%)	(77.3%)	(71.1%)	
	* c)vancomycin*				
8	Aminoglycosides such as gentamicin are very active if they are administered as follows :				
	* a) orally three times daily*	109/179	44/74	65/105	0.741
	*** b) parenteral once daily***	(60.9%)	(59.5%)	(61.9%)	
	* c) parenteral three times daily*				
9	In DR Congo, what is according to your information the estimated resistance rate of *Salmonella* Typhi to Cotrimoxazole (Bactrim)?				
	* a) 0–10%*	76/179	32/75	44/104	0.962
	* b) 10–20%*	(42.5%)	(42.7%)	(42.3%)	
	* c) 25–50%*				
	*** d) 50–75%***				
10	In DR Congo, what is according to your information the estimated resistance rate of *Klebsiella* to Ceftriaxone?				
	* a) 0–10%*	12/173	3/70	9/103	0.258
	* b) 10–20%*	(6.9%)	(4.3%)	(8.7%)	
	* c) 25–50%*				
	*** d) 50–75%***				

* only questions 1–8 were included in the score;

** trimethoprim/sulphamethoxazole.

Less than half and fewer than 10% of respondents correctly estimated the local resistance rate of, *Salmonella* Typhi to trimethoprim-sulphamethoxazole (cotrimoxazole, TMP/SMX) and of *Klebsiella* spp. to ceftriaxone respectively [10], [11]. The other respondents invariably underestimated the resistance rates of both pathogens.

### Awareness

The majority of respondents agreed or strongly agreed that ABR is an important problem, though significantly (p<.0001) less in their own practice as compared to the national or worldwide scope (Table 2). Likewise, most (87.5%) respondents agreed that AB are overused in the country. Factors recognized by respondents as thriving ABR in DRC are listed in Table 3: self-treatment by patients, and non-completing AB treatment were scored by approximately 90% of respondents, but only 69.0% and 71.2% respectively agreed with over-prescription and low quality of AB and less than two-thirds (64.1%) with in-hospital transmission of AB resistant bacteria as thriving factors.

**Table 2:** Perception of Antibiotic resistance as a problem.

Antibiotic resistance is a problem	World wide	In DRC	In my practice
	n (%)	n (%)	n (%)
strongly agree	91 (49.5%)	115 (62.5%)	55 (29.9%)
agree	66 (35.9%)	56 (30.4%)	69 (37.5%)
neutral	16 (8.7%)	8 (4.4%)	20 (10.9%)
do not agree	4 (2.2%)	2 (1.1%)	26 (14.1%)
do not at all agree	6 (3.3%)	3 (1.6%)	13 (7.1%)
no answer	1 (0.5%)	0 (0%)	1 (0.5%)

**Table 3:** Factors contributing to antibiotic resistance in DRC.

	Treatment not completed	Antibiotic not adapted	Too low dosage	Poor quality AB	Too much prescription and consumption	In-hospital transmission	Self-medication
Yes	89.7%	83.2%	82.1%	71.2%	69.0%	64.1%	94.6%
DK*	3.8%	6.0%	8.2%	13.6%	11.4%	12.5%	3.3%
No	4.4%	9.8%	8.2%	10.9%	16.9%	17.9%	1.6%
ND**	2.2%	1.1%	1.6%	4.4%	2.7%	5.4%	0.5%

* Don’t Know.

** No Data.

### Confidence and Seeking Input

Nearly 90% of the respondents declared to feel very confident (n = 21, 11.4%) or confident (n = 142, 77.2%) about their knowledge on AB. Medical students tended to have lower self-confidence in AB prescribing than medical doctors (85.7% versus 94.8%), although this difference did not reach statistical difference (p = .053).There was no significant correlation between confidence and mean knowledge score (p = .1622). Despite this confidence, more than half (55.4%) of respondents agreed with the statement that the selection of the correct AB is difficult. However, when asked about the frequency of consulting a colleague when prescribing AB, most respondents replied sometimes (79.4%) and never (1.1%) versus 3.3%, 11.4% and 4.0% replying half of the times, mostly and always respectively. In line with the lower self confidence among student they also reported to consult significantly more often a colleague compared to medical doctors (25.4% versus 11.6%; p = .019).

### Source of Information

For medical doctors and students combined, the sources of information about AB used during the month prior to the survey were, in rank of decreasing frequency: pharmaceutical companies (consulted by 73.9% of respondents), antibiotic guidelines (66.3%), university courses (63.6%) and information retrieved from the internet (45.7%). Only 26.6% of respondents declared to have used WHO guidelines. Table 4 gives the details for medical doctors versus students: university courses had been used significantly more by students and there were more medical doctors who had consulted internet sources, although this difference did not reach statistical significance. With regard to appreciation of usefulness, all sources were appreciated as useful or very useful by more than 85% of respondents, except for the information provided by pharmaceutical companies (75.5%).

**Table 4:** Sources of information about AB and AB prescribing used in the month prior to the survey, categorized for last year medical students versus medical doctors.

Source of information	Numbers (%) of medical students (n = 106)	Numbers (%) of medical doctors (n = 78)	p
Pharmaceutical companies	76 (71.7%)	60 (76.9%)	0.425
University courses	88 (83.0%)	29 (37.2%)	<.0001
Internet	44 (41.5%)	40 (51.3%)	0.188
Antibiotic guidelines	73 (68.9%)	49 (62.8%)	0.391
WHO guidelines	29 (27.3%)	20 (25.6%)	0.795

### Factors Influencing Prescription

Patient pressure was perceived as a factor contributing to overuse of AB in the community by nearly two-thirds (61.9%) of respondents, whereas only one third (34.3%) did so for the hospital setting (p<.001). The majority of respondents (n = 165; 89.7%) agreed that knowledge of local AB resistance patterns was needed for good prescribing. About two-thirds (63.8%) of respondents did not agree with the statement that choice of prescribed AB was more influenced by availability than by the cause of infection. A total of 72.3% of respondents disagreed with the statement that ABs if not needed do not cause harm to the patient; however, 6.0% were neutral and about one fifth (21.7%) subscribed this statement.

Only 16.3% (n = 30) of respondents agreed that ABs available at the local pharmacies were in most cases of good quality (39.7% mentioned they did not have an opinion about it). One third (30.4%) believed that AB coming through the national central procurement office were of good quality (i.e. significantly higher than those available at the pharmacies; p<.001), whereas 22.8% considered them to be of poor quality and nearly half (44.6%) of respondents did not indicate an opinion.

### Acceptability of Potential Interventions

Nearly three quarters (73.4%) of respondents agreed with the statement that local AB guidelines were more useful than international guidelines. AB committees were only seen by 9.8% of respondents as obstacles rather than as a help, although 42.9% of respondents declared not to have an opinion about this topic. Nearly all (98.8%) respondents expressed their wish for additional training on AB prescribing.

## Discussion

### Limitations and Strengths

KAP-surveys provide insights in the driving forces of AB prescribing and are complementary to surveillance of AB resistance and AB consumption which together constitute baseline information to design interventions about AB prescribing [1]. KAP-surveys however have inherent limitations: for instance, respondents may be triggered to the topic and this - in combination with the multiple choice format of the questionnaire - may direct them to socially desirable answers. Further, expecting a professional in a remote and busy health center to fill in a questionnaire is not evident. Finally, the present survey only addressed medical doctors and students, whereas in practice also nursing staff and traditional healers prescribe ABs in DRC.

To counter these limitations, the present survey was validated by a team of local professionals and took into account limitation in time and concentration of the respondents. In order to avoid influence on answers regarding attitudes and practice questions assessing knowledge were presented at the end of the questionnaire. In order to minimize professional pressure, students were individually addressed by one of their peers (Y.K.). In addition, the questionnaire was distributed and filled-in on-site, precluding consultancy of peers or resources.

Among the strengths of the present KAP-survey was the fact that it addressed most of the topics cited by WHO as influencing AB prescribing, except for economic incentives and workload [1]. By addressing the respondents in an active way, the coverage and response rates were high. Finally, to the best of our knowledge, this is the first survey conducted in a low income country which faces both virtual absence of microbiological facilities and a poor access to quality medicines [12].

### Knowledge

The overall mean score on the knowledge questions (4.9/8 points) was lower than the result from the similar survey conducted in Peru [9] (6.0/7 points). This difference was in large part attributable to the three questions that were worst replied, *i.e.* the questions about AB use in URTI, cross-resistance of MRSA to beta-lactam and AB dose reduction in renal failure. Among the indicators of AB prescribing patterns, WHO has listed the proportions of cases of diarrhea and URTI treated with AB [1]. According to a compilation of community-based studies, the proportion of patients inappropriately receiving AB in both conditions vary between 40–60% [1].Taken into account the present proportion of incorrect answer to the case of URTI (more than two-thirds of respondents would initiate AB treatment) it is clear that this topic is a target for future intervention. Likewise it is striking that nearly two-thirds of respondents did not know about the cross-resistance of MRSA to beta-lactam AB. In part, this may be explained by the absence of microbiological facilities in DR Congo precluding estimates of MRSA incidence [13].

Further, there was insufficient knowledge about local AB resistance rates of *Salmonella* Typhi and *Klebsiella* spp. Although this is not unexpected in a setting with few microbiological facilities, similar findings were observed in the setting in Peru [9] and also in industrialized countries [4].

### Awareness

Similar to previous surveys [5], [6], [8], ABR as a public health problem was found to be highly recognized at the global and national level but less in their own hospital. Among the factors fuelling ABR, patient-related issues (self-medication, not completing treatment) were recognized more frequently as compared to prescriber-related items such as over-prescription. The fact that less than two-thirds of respondents recognized the role of in-hospital spread of AB resistant bacteria is of concern and point to a potential topic of training and sensibilization.

### Confidence and Seeking Input

Despite the fact that more than half of respondents agreed that it is difficult to choose the correct AB, there was high self-confidence in AB prescribing which seemingly contrasted to the modest knowledge scores. Likewise, there was a low propensity to consult colleagues when prescribing AB. Medical students tended to be less self-confident and were more willing to seek input of colleagues. Similar difference in attitudes have been observed between residents (doctors in training) and medical doctors and these observations confirm medical students and junior doctors as a feasible target group for future interventions in AB prescribing [3], [8], [9].

### Source of Information

The availability of unbiased information about AB is a prerequisite for appropriate AB prescribing [1]. For the present setting, several observations were made. First, information from pharmaceutical companies ranked highest in accessibility. Although lowest appreciated for usefulness, it confirms the prominent role pharmaceutical industry as the main source of information in resource- limited settings and this is of concern as drug promotions in these settings may not always be content-directed or evidence-based [14]. By contrast, AB guidelines and in particular WHO guidelines were used less frequently. As expected university courses were significantly more a source of information for medical students than for medical doctors, and this highlights the potential role of university education as a forum for interventions in AB prescribing. In contrast to earlier studies in middle income countries [9], it is important to note that the internet resources were used by less than half of the respondents. This is probably related to the limited access in the country and – as indicated by the difference between use by students versus medical doctors – by economic constraints of access. Future interventions in this setting should acknowledge this and interactive classic face-to-face trainings might be more effective and feasible than e-learning tools in this specific setting.

### Factors that Drive Prescription Practices

Patient pressure was reported as major factor for AB prescription in the DRC although significantly less in the hospital compared to the community setting. In the Peru survey, patient’s demand was even stronger perceived as a thriving factor with more than 70% in the community setting and 50% in the hospital setting. [9].The differences in the overall perception of patient demand as a thriving factor for prescription between the two study sites may be explained in part by the lower economic status of most inhabitants in the Kisangani region. Patients’ demand has been shown to fuel over-prescription especially for URTI in resource rich settings too [15], [16] but may be perceived stronger by the prescribing doctor than actually expressed by the patient [17], [18], [19].

The fact that local AB resistance patterns and the cause of infection rather than the availability of AB were scored as important to the majority of respondents is promising in regard to future interventions. Most of concern however was that more than a quarter of respondents did not contest the statement that unnecessary AB are not of harm to the patient.

AB coming through the national central procurement office [20] were perceived to be of better quality than those available in the local pharmacies. However more than 20% of respondents perceived their quality as not sufficient and 40% did not have an opinion, highlighting the need for quality assurance of drugs and communication to professionals and general public in order to increase confidence.

### Acceptability of and Tracks Potential Interventions

In line with the demand for local AB resistance rates, nearly all respondents welcomed local AB guidelines. AB committees are not yet functioning in DR Congo and this was apparent from the absence of opinions about this topic.

### Future Research and Interventions

The present study shed a light on the AB prescribing behavior by medical doctors and students in resource-limited settings in Central Africa. In order to understand the whole extent of inappropriate AB prescribing and usage, further research is needed among the other channels of AB prescribing and procurement, as well as from private dispensers and the general public. Differences between the results of previous surveys in other countries [4], [5], [6], [7], [8], [9] may advise for campaigns to address the global problem of antibiotic resistance on a local level. From the present survey, potential fields of intervention are the following: improvement of knowledge about AB properties and usage targeting medical students during their university curriculum, the provision of unbiased and evidence based information about AB and local AB resistance rates to all prescribers and the implementation of quality assurance for drugs in order to improve confidence among the general public and professionals. Educational measures such as trainings are highly welcomed, and the implementation of AB committees should be studied at national pilot settings.

## Supporting Information

Table S1 **French and English version of the survey questionnaire.**
(DOC)
