# Supplementary material for: Antibiotic Prescribing in DR Congo: A Knowledge, Attitude and Practice Survey among Medical Doctors and Students
Source: PLoS One. 2013 Feb 18;8(2):e55495. doi: 10.1371/journal.pone.0055495 (PMC3575397; doi:10.1371/journal.pone.0055495)
Supplement: Table S1 — French and English version of the survey questionnaire. (DOC) [file pone.0055495.s001.doc]

| **QUESTIONS (French) / *QUESTIONS (English translation)*** | **REPONSES (French) /*ANSWERS (English translation)*** |
| --- | --- |
| 1. Combien d'années travaillez- vous depuis la fin de vos études de médecine ?  *How many years have you been working after you graduated from medical school?* |  je suis médecin stagiaire (*I am a trainee in medicine)*   moins d’un an (*less than one year)*   1-3 ans (*1- 3 years)*   4-6 ans (*4 – 6 years)*   7 ans et plus (*7 years and more)* |
| 2.Vous prescrivez les antibiotiques à quel type de patients ?  *You are prescribing antibiotics to whom?* |  patients ambulants (*patients at out-patient department)*   patients hospitalisés (*hospitalized patients)*   patients ambulants et hospitalisés (*patients in out-patient department and hospitalised patients)* |
| 3.A quelle fréquence prescrivez-vous des antibiotiques?  *How often do you prescribe antibiotics ?* | □ plus d’une fois par jour (*more than once daily)*  □ une fois par jour *(once daily)*  □ 3-5 fois par semaine (*3 – 5 times a week)*  □ 1-2 fois par semaine (*1 – 2 times a week)*  □ moins d’une fois par semaine (*less than once a week)* |
| 4.La Résistance aux antibiotiques est un problème mondial  *Antimicrobial resistance is a world-wide problem* | □ tout à fait d’accord (*I stronly agree)*  □ d’accord *(I agree)*  □ neutre (pas d’idée) (*neutral)*  □ pas d’accord *(I disagree)*  □ pas du tout d’accord *(I strongly disagree)* |
| 5.La Résistance aux antibiotiques est un problème dans mon pays  *Antimicrobial resistance is a problem in my country* | □ tout à fait d’accord *(I stronly agree)*  □ d’accord *(I agree)*  □ neutre (pas d’idée) (*neutral)*  □ pas d’accord (*I disagree)*  □ pas du tout d’accord (*I strongly disagree)* |
| 6.La Résistance aux antibiotiques est un problème dans ma pratique quotidienne  *Antimicrobial resistance is a problem in my daily practice* | □ tout à fait d’accord *(I stronly agree)*  □ d’accord *(I agree)*  □ neutre (pas d’idée) (*neutral)*  □ pas d’accord (*I disagree)*  □ pas du tout d’accord (*I strongly disagree)* |
| 7.Les antibiotiques sont sur-utilisés dans la communauté en RDC  *Antibiotics are over-used in the community in DRC (the Democratic Republic of the Congo)* | □ tout à fait d’accord *(I stronly agree)*  □ d’accord *(I agree)*  □ neutre (pas d’idée) (*neutral)*  □ pas d’accord (*I disagree)*  □ pas du tout d’accord (*I strongly disagree)* |
| 8.Quelle confiance avez-vous dans votre connaissance des antibiotiques ?  *How confident are you about your knowledge of antibiotics?* | □ très confiant (*very confident)*  □ confiant *(confident)*  □ neutre (pas d’idée) *(neutral (I have no idea))*  □ peu confiant *(a bit confident)*  □ pas du tout confiant *(not at all confident)* |
| 9.A quelle fréquence revoyez-vous votre décision de prescription d’un antibiotique avec un confrère ?  *How many times do you check your decisions about antibiotic prescribing with a colleague ?* | □ jamais *(never)*  □ quelquefois *(sometimes)*  □ la moitié de fois *(half of the times)*  □ la plupart de fois *(mostly)*  □ toujours *(always)* |
| 10.Je crois qu’il est difficile de sélectionner l’antibiotique adapté  *I find it hard to select the correct antibiotic* | □ tout à fait d’accord *(I stronly agree)*  □ d’accord *(I agree)*  □ neutre (pas d’idée) *(neutral)*  □ pas d’accord *(I disagree)*  □ pas du tout d’accord *(I strongly disagree)* |
| 11.Durant l’année écoulée, combien d’enseignements ou des formations avez-vous reçus sur les antibiotiques ?  *During the past years, how many courses or trainings did you receive about antibiotics?* | □ 0  □  1-3  □ 4-6  □ 6-10  □ >10 |
| 12.Parmi les sources d’informations sur les antibiotiques suivantes, lequel avez-vous consulté le mois dernier?  *Among the sources of information about antibiotics listed below, which one did you consult last month?* |  |
| - L’information par les sociétés pharmaceutiques  *- Information supplied by pharmaceutical companies* | □ oui (*yes)*  □ non *(no)* |
| - Informations auprès des cours de l’université  *- Information from University courses* | □ oui (*yes)*  □ non *(no)* |
| - Internet  *- Internet* | □ oui (*yes)*  □ non *(no)* |
| - Guides d’usage d’antibiotiques  *- Antibiotic guidelines* | □ oui (*yes)*  □ non *(no)* |
| - Les guides OMS sur la prise en charge des maladies bactériennes  *- The World Health Organization’s (WHO) guidelines for treatment of bacterial diseases* | □ oui (*yes)*  □ non *(no)* |
| 13.Parmi les sources d’informations sur les antibiotiques suivantes, comment vous les appréciez ?  *How do you appreciate the sources of information about antibiotics listed below:* |  |
| - L’information par les sociétés pharmaceutiques  *- Information provided by pharmaceutical companies* | □ très utile *(very useful)*  □ utile *(useful)*  □ pas du tout utile *(not at all useful)*  □ je ne connais pas *(I do not know)* |
| - Informations auprès d’un confrère  *- Information given by a colleague* | □ très utile *(very useful)*  □ utile *(useful)*  □ pas du tout utile *(not at all useful)*  □ je ne connais pas *(I do not know)* |
| - Informations auprès des cours de l’université  *- Information from University courses* | □ très utile *(very useful)*  □ utile *(useful)*  □ pas du tout utile *(not at all useful)*  □ je ne connais pas *(I do not know)* |
| - Internet  *- Internet* | □ très utile *(very useful)*  □ utile *(useful)*  □ pas du tout utile *(not at all useful)*  □ je ne connais pas *(I do not know)* |
| - Guides d’usage d’antibiotiques  *- Antibiotic guidelines* | □ très utile *(very useful)*  □ utile *(useful)*  □ pas du tout utile *(not at all useful)*  □ je ne connais pas *(I do not know)* |
| -Les guides OMS sur la prise en charge des maladies bactériennes  *- The World Health Organization’s (WHO) guidelines for treatment of bacterial diseases* | □ très utile *(very useful)*  □ utile *(useful)*  □ pas du tout utile *(not at all useful)*  □ je ne connais pas *(I do not know)* |
| 14.Quand on prescrit un antibiotique, il est important de connaître le taux de résistance de bactéries qu’on trouve localement  *When one prescribes an antibiotics, it is important to know the resistance rate of the bacteria in the local setting* | □ tout à fait d’accord *(I stronly agree)*  □ d’accord *(I agree)*  □ neutre (pas d’idée) *(neutral)*  □ pas d’accord *(I disagree)*  □ pas du tout d’accord *(I strongly disagree)* |
| 15.Mon choix pour la prescription d’antibiotiques est plus orienté par la disponibilité des antibiotiques que par la cause de l’infection  *My choice for prescribing antibiotics is more influenced by the availability of antibiotics than by the cause of the infection* | □ tout à fait d’accord *(I stronly agree)*  □ d’accord *(I agree)*  □ neutre (pas d’idée) *(neutral)*  □ pas d’accord *(I disagree)*  □ pas du tout d’accord *(I strongly disagree)* |
| 16.La demande des patients pour des antibiotiques contribue à leur sur-utilisation au sein de la communauté  *Patients’ demands for antibiotics contribute to the overuse of antibiotics in the community* | □ tout à fait d’accord *(I stronly agree)*  □ d’accord *(I agree)*  □ neutre (pas d’idée) *(neutral)*  □ pas d’accord *(I disagree)*  □ pas du tout d’accord *(I strongly disagree)* |
| 17.La demande des patients pour des antibiotiques contribue à leur sur-utilisation au sein de l’hôpital  *Patients’ demands for antibiotics contribute to the overuse of antibiotics in the hospital* | □ tout à fait d’accord *(I stronly agree)*  □ d’accord *(I agree)*  □ neutre (pas d’idée) *(neutral)*  □ pas d’accord *(I disagree)*  □ pas du tout d’accord *(I strongly disagree)* |
| 18.Je pense que certains antibiotiques disponibles localement sont de faible qualité  *I think that antibiotics that are available locally are of bad quality* | □ tout à fait d’accord *(I stronly agree)*  □ d’accord *(I agree)*  □ neutre (pas d’idée) *(neutral)*  □ pas d’accord *(I disagree)*  □ pas du tout d’accord *(I strongly disagree)* |
| 19.Le développement de lignes directrices locales (guidelines locaux) serait plus utile que les lignes directrices internationales  *For antibiotic guidelines local guidelines are more useful than international guidelines* | □ tout à fait d’accord *(I stronly agree)*  □ d’accord *(I agree)*  □ neutre (pas d’idée) *(neutral)*  □ pas d’accord *(I disagree)*  □ pas du tout d’accord *(I strongly disagree)* |
| 20.Les lignes directrices (guidelines) sur les antibiotiques et les comités antibiotiques sont plus des obstacles qu’une aide  *Antibiotic guidelines and antibiotic committees are rather obstacles than a help* | □ tout à fait d’accord *(I stronly agree)*  □ d’accord *(I agree)*  □ neutre (pas d’idée) *(neutral)*  □ pas d’accord *(I disagree)*  □ pas du tout d’accord *(I strongly disagree)* |
| 21.Je souhaiterais l’organisation de programme de formation sur les antibiotiques  *I welcome the implementation of a training program about antibiotics* | □ tout à fait d’accord *(I stronly agree)*  □ d’accord *(I agree)*  □ neutre (pas d’idée) *(neutral)*  □ pas d’accord *(I disagree)*  □ pas du tout d’accord *(I strongly disagree)* |
| 22.Je crois que la prescription d’antibiotique si le patient n’en a pas besoin n’est pas préjudiciable  *I think that prescribing antibiotics if the patient does not need them does not cause harm to the patient* | □ tout à fait d’accord *(I stronly agree)*  □ d’accord *(I agree)*  □ neutre (pas d’idée) *(neutral)*  □ pas d’accord *(I disagree)*  □ pas du tout d’accord *(I strongly disagree)* |
| 23.Un fillette de 4 ans vient consulter pour diarrhée depuis 4 jours (3 exonérations par jour). Elle n’a pas fait de fièvre dans son histoire et elle est afébrile. Quelle est votre conduite à tenir?  *A 4-year-old child had diarrhea since 4 days (3 stools daily). She had no fever during the past days nor at consultation. What is your treatment choice?* | □ amoxicilline po *(amoxicillin orally)*  □ bactrim po *(trimethoprim/sulphamethoxazole orally)*  □ coamoxiclav po *(amoxicillin/clavulanic acid orally)*  □ aucun antibiotique, juste une réhydratation orale *(no antibiotic, just oral rehydration salts)* |
| 24.Un enfant 6 ans a la fièvre à 38 °C, sécrétions nasales et mal de gorge depuis deux jours. A l’inspection de la gorge on note une rougeur. Quelle thérapie est-ce que vous recommandez?  *A 6-year-old child has fever (38°C), nasal discharge and a painful throat for two days. At visual inspection, the throat is reddish. What is your treatment choice?* | □ bactrim po *(trimethoprim/sulphamethoxazole orally)*  □ amoxicilline po *(amoxicillin orally)*  □ coamoxiclav po *(amoxicillin/clavulanic acid orally)*  □ aucun antibiotique *(no antibiotic)* |
| 25.Pendant votre tour de salle, vous voyez deux malades avec insuffisance rénale sévère.  Le Patient A est un homme de 68 ans avec une cellulite sérieuse de la jambe. Il est sous clindamycine  Le patient B est une femme diabétique de 64 ans sous ceftriaxone comme antibiothérapie à l’aveugle (empirique) pour septicémie.  La réduction de la dose est nécessaire chez ?  *During ward round, you have seen two patients with impaired renal function. Patient A is a 68 year-old male with cellulitis in the lower limb. He is administered clindamycin. Patient B is a 64 year-old woman with diabetes who received empirically treatment for sepsis with ceftriaxone. In which case you will need to adjust the antibiotic dose?* | □ patient A *(patient A)*  □ patient B *(patient B)*  □ patient A et B *(patient A and B)*  □ ni chez le patient A, ni chez le patient B *(Neither patient A nor patient B)* |
| 26.Lequel des antibiotiques suivants peut être donné sans risque pendant le premier trimestre de grossesse  *Which one of the following antibiotics may be safely given during the first trimester of pregnancy?* | □ amoxicilline *(amoxicillin)*  □ ciprofloxacine *(ciprofloxacin)*  □ gentamicine *(gentamicin)* |
| 27.Lequel des antibiotiques suivants a la meilleure activité contre les bactéries anaérobies  *Which of the following antibiotics has the best activity against anaerobes?* | □ ciprofloxacine *(ciprofloxacin)*  □ metronidazole *(metronidazole)*  □ bactrim *(trimethoprim/sulphamethoxazole)* |
| 28.Le Staphylocoque aureus résistant à la méthicillin est sensible à:  *Methicillin resistant - Staphylococcus aureus is susceptible to:* | □ co-amoxiclav  *(amoxicillin clavulanic acid)*  □ cefotaxime *(cefotaxime)*  □ ceftriaxone *(ceftriaxone)*  □ aucun de ces antibiotiques *(none of these antibiotics)* |
| 29. Lequel des antibiotiques suivants est pénètre le plus efficacement dans le cerveau (traverse la barrière hémato-méningé)  *Which of the following antibiotic most effectively crosses the blood-brain barrier?* | □ clindamycine *(clindamycin)*  □ ceftriaxone *(ceftriaxone)*  □ vancomycine *(vancomycin)* |
| 30.Les aminoglycosides tels que gentamicine sont très actifs s’ils sont administrés  *Aminoglycoside antibiotics such as gentamicin are most active when they are administered as follows :* | □ par voie orale, trois fois quotidiennement *(orally, three times daily)*  □ par voie parentérale, une fois quotidiennement *(parenterally, once daily)*  □ par voie parentérale, trois fois quotidiennement *(parenterally, three times daily)* |
| 31.En RDC, quel est à votre estimation le taux de résistance de Salmonella Typhi à la cotrimoxazole (Bactrim) ?  *What is the rate of resistance of Salmonella Typhi to trimethoprim/sulphamethoxazole in the Democratic Republic of the Congo?* | □ 0 – 10%  □ 10 – 20%  □ 25- 50%  □ 50 – 75% |
| 32.En RDC, quel est à votre estimation le taux de résistance de Klebsiella à la ceftriaxone ?  What is the rate of resistance of *Klebsiella pneumoniae* to trimethoprim/sulphamethoxazole in the Democratic Republic of the Congo? | □ 0 – 10%  □ 10 – 20%  □ 25- 50%  □ 50 – 75% |
| 33. Dans la plupart de cas, les antibiotiques disponibles par les pharmacies sont des produits d’une bonne qualité  *In most of the cases, the antibiotics available in local pharmacies are of good quality* | □ oui *(yes)*  □ non *(no)*  □ je ne sais pas *(I do not know)* |
| 34.Dans la plupart de cas, les antibiotiques disponibles par les centrales d’achat sont de bonne qualité  *In most cases, the antibiotics available in the central procurement stores are of good quality* | □ oui *(yes)*  □ non *(no)*  □ je ne sais pas *(I do not know)* |
| 35.Quel facteur parmi les suivant contribue(nt) à la résistance aux antibiotiques en RDC  *Among the factors listed below, which one(s) contribute(s) to antimicrobial resistance in the Democratic Republic of the Congo:* |  |
| - le malade ne finit pas son cure  *- the patient does not finish his/her treatment* | □ oui *(yes)*  □ non *(no)*  □ je ne sais pas *(I do not know)* |
| - les antibiotiques ne sont pas adaptés au germe responsable  *- the antibiotics are not adapted to the bacterium that causes the infection* | □ oui *(yes)*  □ non *(no)*  □ je ne sais pas *(I do not know)* |
| - les antibiotiques sont sous-dosés  *- the antibiotics are given at a dose which is too low* | □ oui *(yes)*  □ non *(no)*  □ je ne sais pas *(I do not know)* |
| - les antibiotiques sont de mauvaise qualité  *- the antibiotics are of bad quality* | □ oui *(yes)*  □ non *(no)*  □ je ne sais pas *(I do not know)* |
| - on prescrit trop vite des antibiotiques et on consomme trop  *- there is too much prescription and too much consumption of antibiotics* | □ oui *(yes)*  □ non *(no)*  □ je ne sais pas *(I do not know)* |
| - les bactéries résistantes sont transmises dans les hôpitaux  *- resistant bacteria are transmitted in the hospitals* | □ oui *(yes)*  □ non *(no)*  □ je ne sais pas *(I do not know)* |
| - automédication par les malades  *- self medication by the patient* | □ oui *(yes)*  □ non *(no)*  □ je ne sais pas *(I do not know)* |
